# Ultrasound‐guided core needle biopsy combined with immunohistochemistry and molecular testing improve the diagnostic accuracy of bone metastases from follicular thyroid carcinoma, two case reports and analyses

**DOI:** 10.1002/ccr3.8959

**Published:** 2024-05-29

**Authors:** Zhiyuan Li, Jianbin Su, Jinjing Wang, Li Yan, Huiqiang Zhang, Xinyu Li, Yanhong Tai, Yi Fang, Tao Yan

**Affiliations:** ^1^ Outpatient Department Fifth Medical Center of Chinese PLA General Hospital Beijing China; ^2^ Department of Endocrinology and Metabolism Fifth Medical Center of Chinese PLA General Hospital Beijing China; ^3^ Center for Disease Control and Prevention of Xicheng District Beijing China; ^4^ Department of Pathology Fifth Medical Center of Chinese PLA General Hospital Beijing China

**Keywords:** *BRAF*‐like malignancies, core needle biopsy, follicular thyroid carcinoma, immunohistochemistry, *RAS*‐like malignancies, spinal metastases

## Abstract

**Key Clinical Message:**

Ultrasound‐guided core needle biopsy combined with immunohistochemistry and molecular testing could improve the diagnostic accuracy of bone metastases from follicular thyroid carcinoma, help to predict distant metastasis and prognosis.

**Abstract:**

Metastatic thyroid follicular carcinoma presenting initially with bone lesion is uncommon, its prime symptom is gradual onset, localized pain. Patient with bone metastasis who were diagnosed before thyroidectomy had a higher rate of mortality, clinician should be cautious in eliciting the clinical history and this insidious symptom in middle age group, carry out further examination. We are presenting two case reports of a follicular thyroid carcinoma with bone metastasis, ultrasound‐guided core needle biopsy combined with immunohistochemistry (IHC) were carried out by our clinical team to determine the source and nature of the tumor, relevant literature was reviewed, molecular testing was discussed, we believe core needle biopsy combined with IHC and molecular testing improve the diagnostic accuracy of bone metastases from follicular thyroid carcinoma.

## 1 INTRODUCTION

Follicular thyroid carcinoma (FTC) accounts for 10%–20% of thyroid malignancies, with a higher incidence in women over 40 years of age, which is the second most common differentiated thyroid cancer (DTC) after papillary thyroid carcinoma (PTC).^1^ Follicular thyroid cancer is characterized by hematogenous metastasis via blood stream to distant organs and most common sites of metastatic lesions include lung, bone, brain, or liver.^2^ Bone metastasis is found most frequently in organs of high blood flow, such as the red marrow regions of the axial skeleton, it causes the occurrence of pain, pathological fracture, spinal cord compression and hypercalcemia, and affects the quality of life.^3^ We found that the current literature and evidence has not clarified the optimal biopsy technique for the diagnosis of such tumors. we report two rare cases of bone metastases from follicular thyroid carcinoma, distant metastases are sole initial manifestation, ultrasound‐guided core needle biopsy (CNB) combined with immunohistochemistry (IHC) were carried out to determine the source and nature of the tumor, relevant literature was reviewed to analyze and discuss the diagnostic accuracy and advantage CNB, clinical utility of molecular testing for prognoses of follicular thyroid carcinoma is discussed as well.

## 2 THE FIRST CASE REPORT

### 2.1 Case history and examination

**FIGURE 1:**
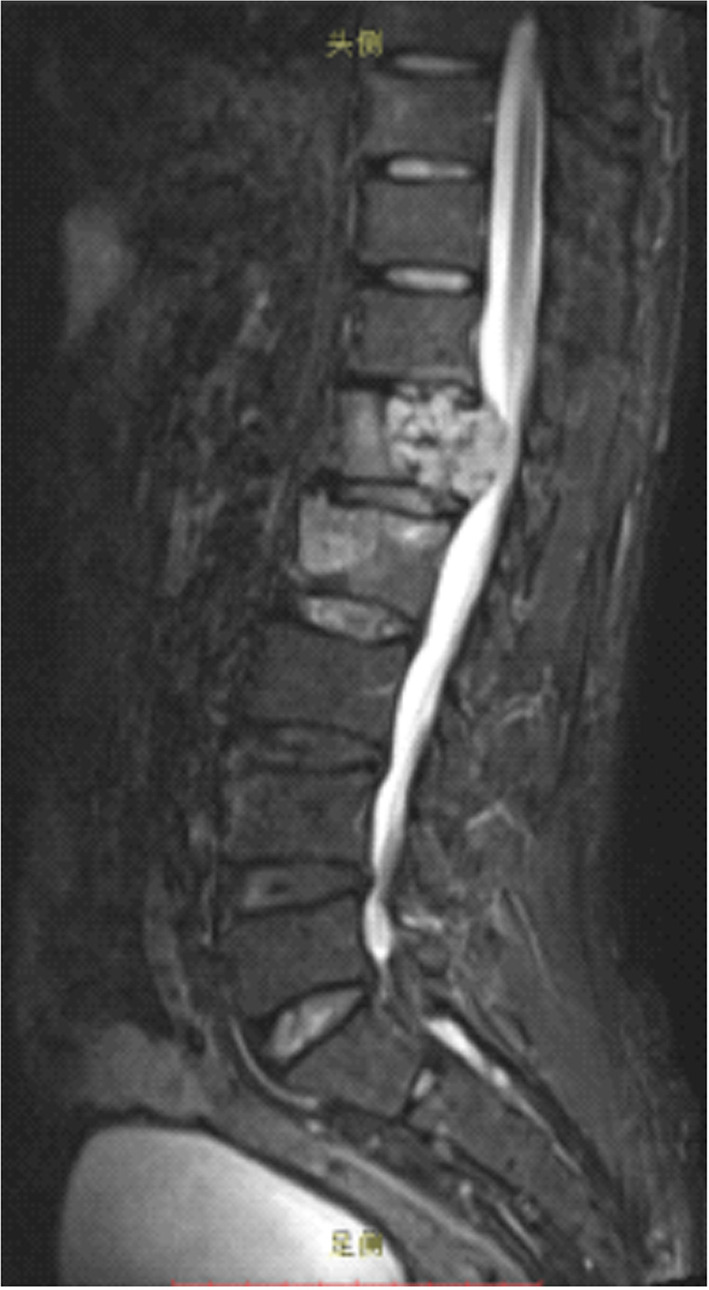
T2 sagittal MRI of the spine in a 57‐year‐old male, it showed lesions with signal abnormalities in L1 and L2 vertebrae, lumbar spinal canal stenosis with cord compression caused by extended lesion at L1 level.

**FIGURE 2:**
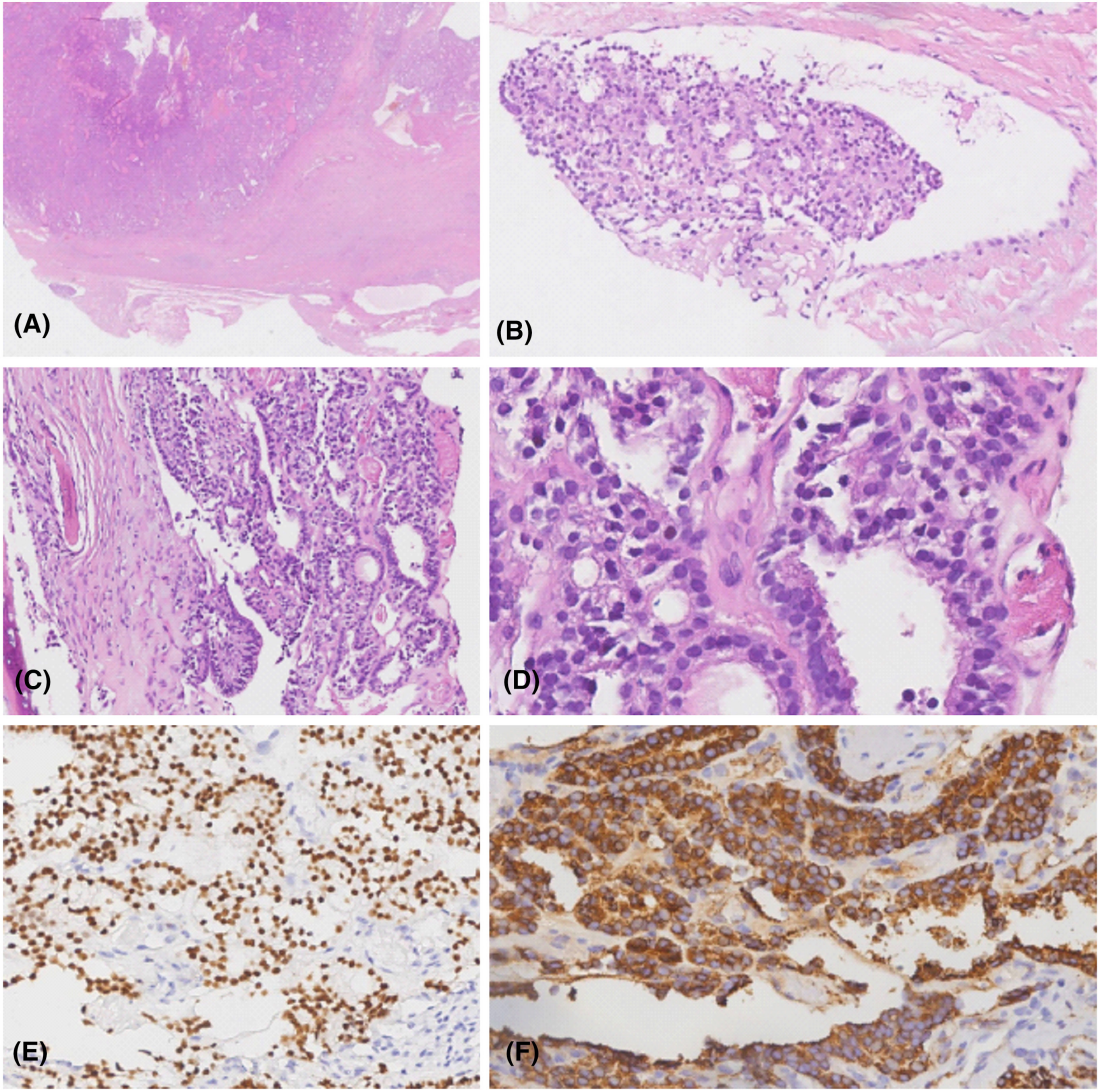
Histological appearance of follicular thyroid carcinoma with spinal metastasis in a 57‐year‐old male. Photomicrograph shows follicular thyroid carcinoma, tumor tissue invasion was observed in the capsule vessels (not within the tumor) in left thyroid lobe from thyroidectomy specimen (A) and (B), neoplastic follicular type infiltration was observed in bone tissue from the L1 vertebra lesion after ultrasound‐guided core needle biopsy, consistent with a metastatic follicular thyroid carcinoma (C) and (D). (H&E stain, original magnification, 10× in A, 200× in B and C, and 400× in D). IHC reveals follicular cells in bone tissue were positive staining for TTF‐1 (E) and TG (F).

Our first patient is a 57‐year‐old man, who had a 6 months history of progressive lower back pain and bilateral hip pain, which was of gradual onset, not sharp in nature. When he sought care at local hospital, magnetic resonance imaging (MRI) showed lesions with signal abnormalities in L1 vertebrae, its left pedicle, and L2 vertebrae, lumbar spinal canal stenosis with cord compression caused by extended lesion at L1 level (Figure 1). Systemic scans were performed after IV administration of Technetium‐99 m methylene diphosphonate (^99m^Tc‐MDP), there was accumulation of radioactivity within L1 and L2 vertebra. Ultrasound‐guided core needle biopsy was performed at the L1 vertebra lesion and tissue was sent for histopathological examination. Neoplastic follicular type infiltration was observed in the bone tissue, consistent with a metastatic follicular thyroid carcinoma (Figure 2C,D). IHC was performed using a panel of antibodies to tumor markers, which revealed follicular cells were positive staining for thyroid transcription factor‐1 (TTF‐1), thyroglobulin (Tg) (Figure 2E,F), cytokeratin (CK), CK8/18 and weakly positive for CK7, while negative for calcitonin, excluding medullary thyroid cancer. Then retrograde evaluation for a primary thyroid malignancy had been made. Color Doppler ultrasound demonstrated a multinodular thyroid gland, a 3.0 cm × 1.8 cm solid hypoechoic nodule was noted in the left lobe with punctate echogenic foci, unclear margin, and regular shape. At this stage, the patient underwent total thyroidectomy plus central lymph node dissection for histopathological confirmation and staging of cancer. The histopathology of the left thyroid lobe revealed follicular thyroid carcinoma, tumor tissue invasion was observed in the capsule vessels (not within the tumor) in thyroidectomy specimen (Figure 2A,B) and cystic lesion, absence of metastatic lymph nodes in the central compartment. Thus, diagnosis of metastatic follicular carcinoma from thyroid origin of these lumbar vertebra lesions was made based on histopathology, IHC and imaging findings. The tumor was staged as pT3aN0M1.

### 2.2 Investigations, treatment, and follow‐up

**FIGURE 3:**
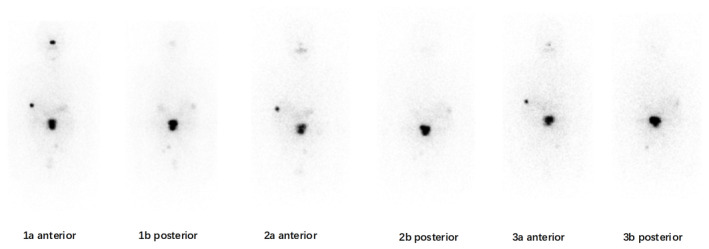
Whole‐body scintigraphy images obtained after the first (1a, 1b), second (2a, 2b), and third (3a, 3b) administration of radioiodine (I‐131) therapy with 200 mCi each time in a 57‐year‐old male with spinal metastatic follicular thyroid carcinoma. The consecutive whole‐body scans demonstrated multiple foci of I‐131 uptake in the L1 and L2 vertebra, right sixth rib, there were no significant changes during the clinical course. The whole‐body scintigraphy findings were consistent with the concomitant high serum concentration of thyroglobulin.

A month after the operation without endocrine suppression treatment, he was referred to Endocrine and Metabolic Department in our hospital to receive radioactive iodine treatment. His thyroid function test on admission showed the following values: thyroid stimulating hormone (TSH): 119.63 (0.55–4.78) μIU/mL, free T_3_: 1.3 (2.8–6.3) pmol/L and free T_4_ < 1.3 (11.5–22.7) pmol/L. Serum thyroglobulin's levels was 1426.00 (3.5–77) ng/mL, while anti‐thyroglobulin antibody (Tgab) was within the normal range. Serum biochemistry showed impaired fasting glucose and hyperlipidemia. The ultrasound examination of the neck showed postoperative alterations in the thyroid bed, absence of abnormal lymph nodes. Computed tomography (CT) showed no evidence of metastatic lesion in the lung. Our patient took radioactive iodine (RAI) therapy with a dose of I‐131 7.4 GBq (200 mCi), a whole‐body radioactive iodine (I‐131) scintigraphy on post‐therapy days three indicated multiple foci of increased I‐131 uptake in the L1 and L2 vertebra, right sixth rib, without any uptake in the brain, lung, liver, and thyroid bed (Figure 3). He took thyroid hormone replacement therapy (levothyroxine 125 μg/day) after the procedure, dose was adjusted to maintain the TSH concentration under 0.1 μIU/mL and proper serum thyroid hormones. During his hospitalization, his low back pain gradually worsened, then our patient was transferred to the Department of Orthopedics within our hospital, he underwent partly excision of the metastasis lesions, laminectomies, and internal fixation at L1 and L2 vertebra. The patient made a good symptomatic recovery and his low back pain improved postoperatively. Our patient received the second and third RAI therapy with a dose of I‐131 7.4 GBq each time. Tg level of our patient gradually decreased to 1205 ng/mL without endocrine suppression treatment before the second RAI therapy, it elevated to 2085 ng/mL before the third RAI therapy. The I‐131 whole‐body scan demonstrated multiple foci of positive functional metastases, L1 and L2 vertebra, right sixth rib, persisted without significant alternation during the clinical course (Figure 3). The whole‐body scintigraphy findings were consistent with the concomitant high serum concentration of thyroglobulin. Our patient did not respond to I‐131 therapy, then he switched to target chemotherapy.

## 3 THE SECOND CASE REPORT

### 3.1 Case history and examination

Our second case is a 68‐year‐old female, she was presented to a local clinic for help due to self‐detected lump on her right shoulder, and she had suffered from an isolated intermittent pain on her right shoulder for 9 months. She had a history of nodule resection in the right lobe of her thyroid 4 years ago, followed pathological findings showed a benign lesion. She had been already evaluated with supplementary examinations in a local hospital: thyroid ultrasonography revealed enlarged bilateral lobes and isthmus with their irregular shape, multiple hypoechoic nodules with defined boundary and regular shape. The largest nodule was approximately 1.71 × 1.55 cm in right lobe and 3.21 × 2.51 cm in left lobe with heterogeneous echo pattern, blood flow signals were observed inside the nodules and surrounding tissue. MRI of the shoulder showed an occupying lesion in her right spealbone. ^99m^Tc‐MDP whole body bone scan revealed increased uptake noted in the right cranial bone, right humerus, and right scapula bone, considering bone metastatic lesion. Whole‐body positron emission tomography computed tomography (PET/CT) scan demonstrated positive uptake in left lobe of thyroid gland, numerous pulmonary nodules, multiple enlarged lymph nodes within mediastinum, osteolytic destruction in right scapula and right cranial bone.

### 3.2 Investigations, treatment, and follow‐up

**FIGURE 4:**
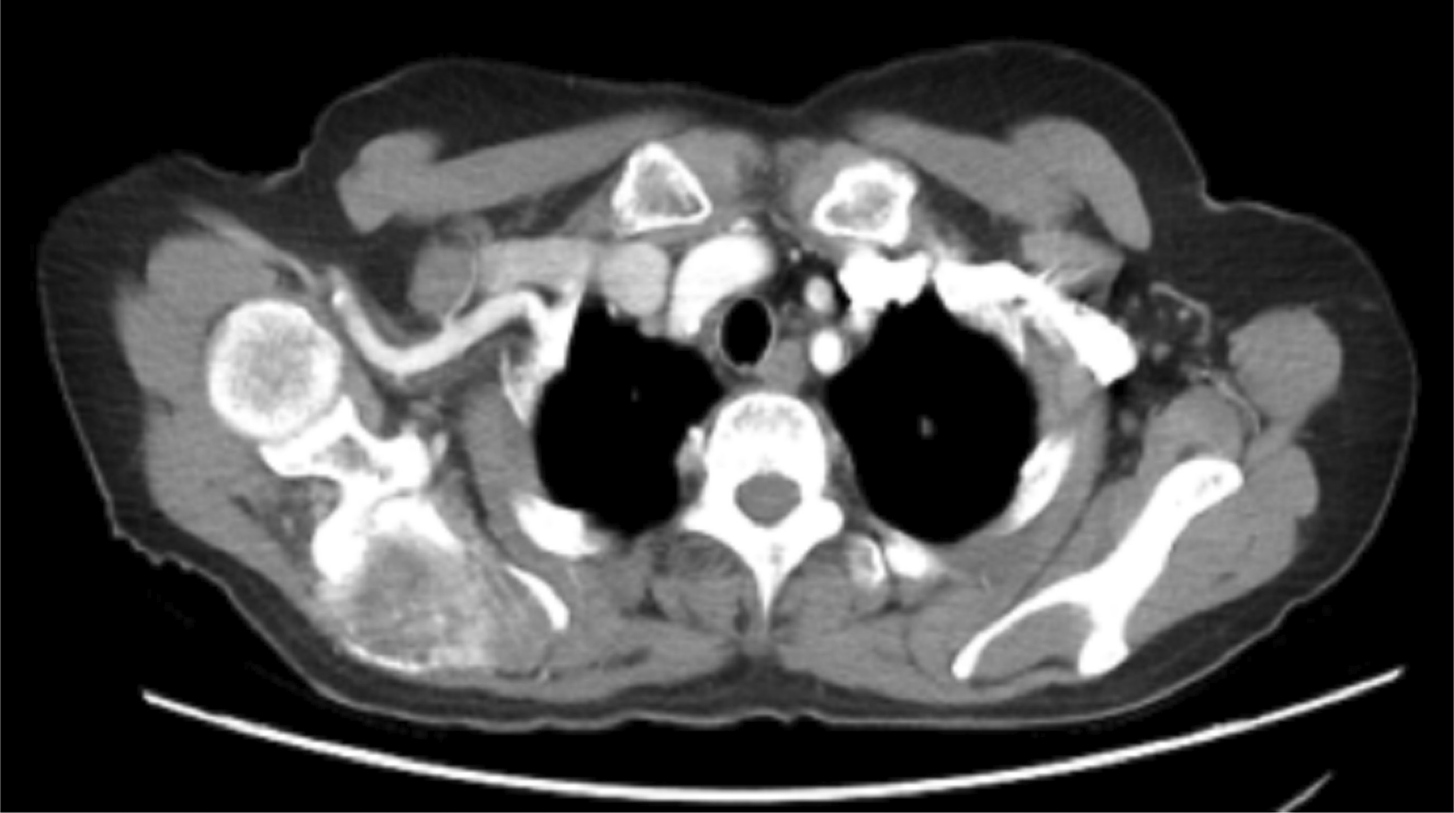
CT image of the chest in a 68‐year‐old female, it revealed osseous destruction and soft tissue mass in the right scapula, suggestive of bone tumor.

**FIGURE 5:**
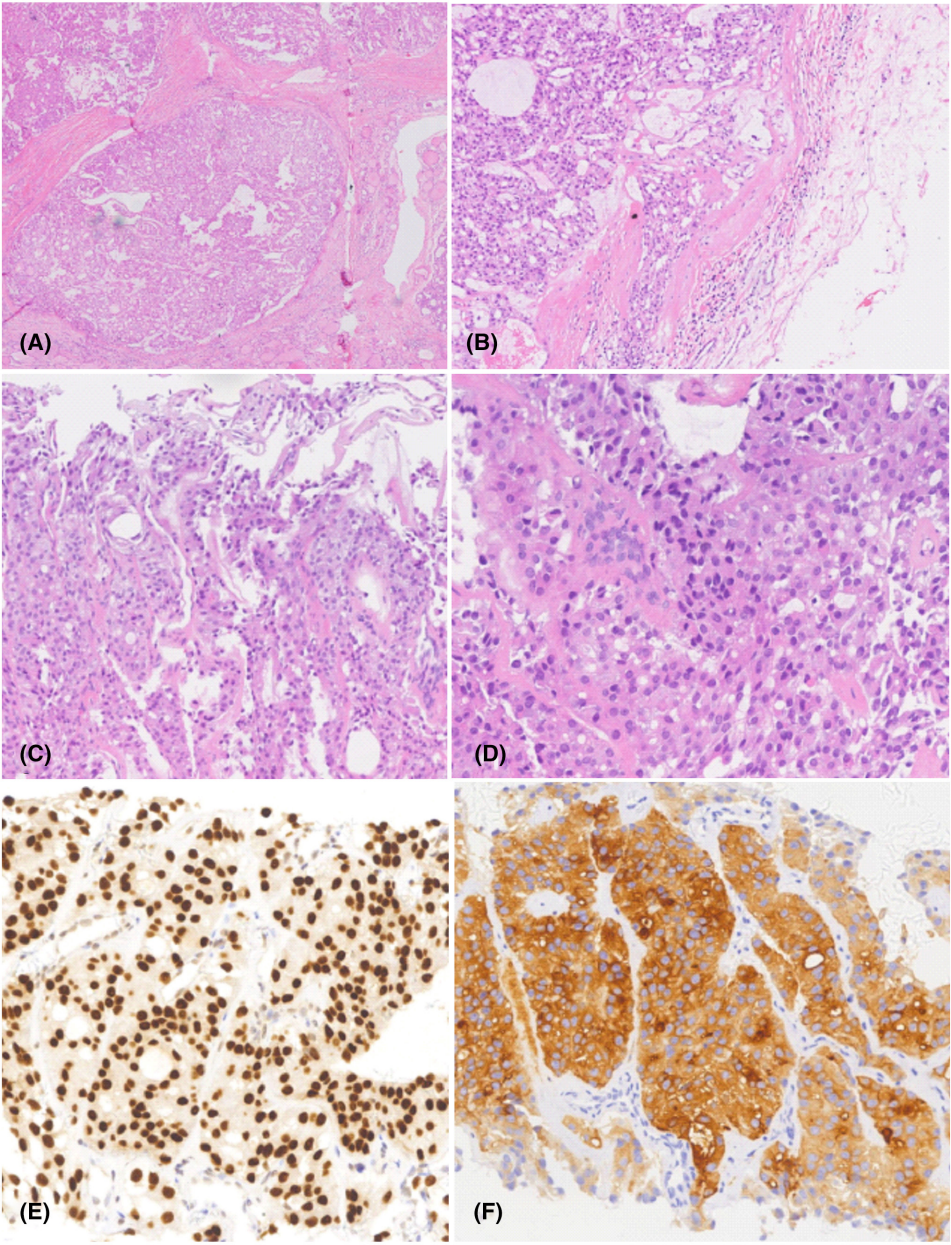
Histological appearance of follicular thyroid carcinoma with right spealbone metastasis in a 68‐year‐old female. Photomicrograph reveals follicular thyroid carcinoma with capsular infiltration in left lobe from thyroidectomy specimen (A) and (B), core needle biopsy revealed epithelioid neoplastic cells in a nest‐like in spealbone lesion (C) and (D). (H&E stain, original magnification, 100× in A, B, and 200× in C, D). IHC reveals follicular cells in bone tissue were positive staining for TTF‐1, TG, (E) and (F).

**FIGURE 6:**
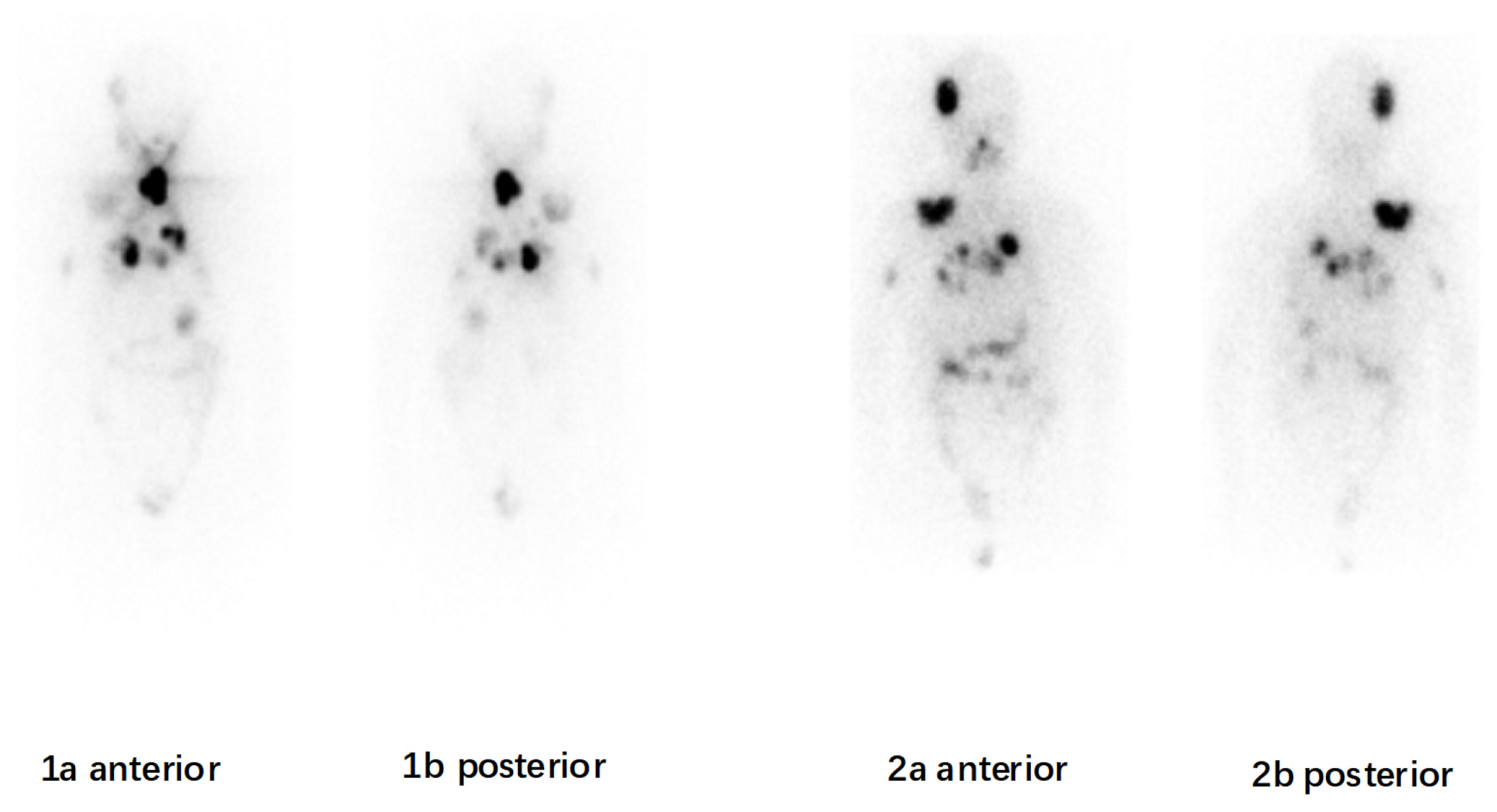
Whole‐body scintigraphy images obtained after the first (1a, 1b), second (2a, 2b) administration of radioiodine (I‐131) therapy with 200 mCi each time in a 68‐year‐old female with right spealbone metastatic follicular thyroid carcinoma. The first WBS indicated multiple foci of increased I‐131 uptake in the right cranium, thyroid bed, chest, right spealbone after the first therapy. The second WBS indicated remnant uptake in thyroid bed disappeared, multiple foci metastasis in chest persisted. Osseous destruction in right cranium and right spealbone metastases developed into large masses. The whole‐body scintigraphy findings were further validated by the concomitant high serum concentration of thyroglobulin.

She was suspected of being diagnosed with bone metastases from thyroid carcinoma and referred to our hospital for further evaluation. Upon physical examination, local examination of the neck revealed a 4 × 3 cm swelling, arising from the left lobe of the thyroid, the mass on her right shoulder was solid, fixed with tenderness, around 9 × 10 cm in size. Computed tomography image of the chest revealed osseous destruction and soft tissue mass in the right scapula (Figure 4) and multiple lung metastases. Ultrasound‐guided core needle biopsy on her right shoulder was performed and revealed epithelioid neoplastic cells in a nest‐like pattern (Figure 5C,D). IHC was positive for TTF‐1, Tg (Figure 5E,F), CK, PAX8 (a member of paired‐box transcription factors), and weakly positive for CK7, establishing her thyroid as the primary source of the bone lesion. Surgery was recommended to the patient and she underwent total thyroidectomy in our hospital, and then postoperative pathology hematoxylin and eosin (H&E) staining revealed a 3.4 × 3 × 2.1 cm follicular thyroid carcinoma in the left lobe with capsular infiltration (Figure 5A,B) and absence of metastatic lymph nodes in the central compartment. *BRAF* p.V600E mutation testing was wild type. Our patient proceeded to undergo radiofrequency ablation (RFA) procedures for right scapula and right cranial bone lesions for local tumor control. Three weeks later without endocrine suppression treatment she was readmitted and treated with an oral dose of 200 mCi radioactive iodine (I‐131). Her Tg level was 28,305 ng/mL, while Tgab was within the normal range (TSH > 150 μIU/mL). The whole‐body radioactive iodine (I‐131) scintigraphy indicated multiple foci of increased I‐131 uptake in the right cranium, thyroid bed, chest, and right spealbone (Figure 6). The patient underwent the second RAI therapy with the same dose 6 months after the first therapy, her Tg level (TSH > 150 μIU/mL) increased to 40,415 ng/mL, the whole‐body radioactive iodine (I‐131) scintigraphy indicated remnant uptake in thyroid bed disappeared, multiple foci metastasis in chest persisted, right cranium and right spealbone metastases revealed osseous destruction, developed into large masses (Figure 6). She switched to target chemotherapy, her endocrine suppression treatment was satisfactory.

## 4 DISCUSSION

Most of thyroid carcinoma presents initially as painless nodule, often discovered incidentally by ultrasonography, very rare cases of differentiated thyroid cancer manifest as distant metastasis at the time of initial diagnosis.^4^ The prime presenting symptom of bone metastasis is gradual onset of localized pain, it often occurs at night or pain when recumbent, it may relieve with activity or NSAID (Nonsteroidal Anti‐inflammatory Drugs).^5^ Back pain is a common complaint and classic feature of spine malignancy, which is caused by periosteal stretching and inflammation.^5^ Spinal metastasis may also manifest as radicular pain depending on the tumor infiltration pattern. Patients present with back pain who are diagnosed with bone metastasis before thyroidectomy has a higher rate of mortality,^6^ therefore, clinician should be cautious in eliciting the clinical history and this insidious symptom in middle age group, carry out further imaging examination.

The diagnosis of primary or metastatic bone neoplasms is difficult and challenging, pretreatment biopsy is a key step, we found that the current literature and evidence has not clarified the optimal biopsy technique for the diagnosis of such tumors.^7^ Some studies designed to evaluate the accuracy of CNB and FNA. A retrospective study with 405 specimens of 389 patients analyzed the accuracy rates of FNA and CNB procedures, the accuracy rates of FNA and CNB were 86.6% and 93.8%, respectively. Both FNA and CNB procedures had high accuracy rates. The image‐guided procedure had a low sampling error rate in cases with no palpable masses, which was an effective method for obtaining tissue samples.^7^ Another prospective study in patients above 10 years old compared diagnostic accuracy in 50 consecutive concurrent needle core biopsies and fine‐needle aspirations of musculoskeletal lesions, in all aspects, including determining the nature of the tumor, establishing the histologic type and grade, and achieving a specific diagnosis, the CNB had a higher diagnostic accuracy than fine‐needle aspiration.^8^ A large study including several hundred cases achieved a diagnostic accuracy of 89% of CNB in 509 cases of bone and soft tissue tumors, it is easily available and less invasive than incisional biopsy.^9^ Another advantage of CNB, it avoids the risk of destabilizing an already diseased spinal or peripheral skeleton segment, while image guided CNB additionally provides immediate confirmation of the correct needle location inside the target area.

IHC is useful to identify and visualize presence of specific protein markers through light microscopy, assist with accurate tumor classification and diagnosis. Thyroid transcription factor‐1 is a tissue‐specific transcription factor mainly expressed in thyroid follicular cells and alveolar epithelial cells. Thyroglobulin is a tissue specific‐protein produced by thyroid follicular cells, it is a thyroid carcinoma marker used in diagnostic pathology.^10^, ^11^ IHC staining of the CNB of our patient was positive for TTF‐1 and Tg, with no lung lesion visualized on imaging studies, confirming thyroid as the primary source of these bone lesions.

The occurrence of differentiated thyroid carcinoma is a multigene and multistep carcinogenesis process, specific mutually exclusive molecular alterations can promote tumor evolvement and start a multistep tumorigenic process. There is a close correlation between genetic changes and histopathological features,^12^, ^13^ the major genetic changes in conventional PTC are *BRAF* p.V600E, *RET/PTC*, *NTRK*, and *ALK* rearrangements. *BRAF* p.V600E mutation exists in about conventional 50% of PTCs, which is a useful diagnostic marker. It has been linked to extrathyroid tumor extension and to an increased risk of PTC recurrence. In FTC, *RAS* point mutation, *TERT* promoter mutation and *PAX8‐PPARγ* fusion are the main genetic abnormalities.^14^, ^15^ Some study suggests that *RAS*‐mutated FTC may relate to a poor prognosis^16^ or distant metastasis.^17^ *TERT* promoter mutations are associated with *RAS* mutations in a subset of FTC, it has been linked to poor prognosis in FTC.^18^ The 5th edition of 2022 WHO Classification of Thyroid Neoplasms has divided thyroid tumors into several new categories according to clearer understanding of the cell of origin, pathologic features, molecular classification, and biological behavior.^19^ Papillary thyroid carcinoma with many morphological subtypes, represent the *BRAF*‐like malignancies, whereas invasive encapsulated follicular variant PTC and follicular thyroid carcinoma represent the *RAS*‐like malignancies.^20^ The current classification emphasizes the value of biomarkers that may aid diagnosis and provide prognostic information.

For patients with extrathyroidal extension or distant metastatic sites, total thyroidectomy is required to remove all primary neoplasm, allows subsequent treatment of metastatic disease with RAI.^21^ Its therapeutic effects are evaluated based on alteration in serum Tg and changes in anatomical imaging of bone metastatic lesions. Our two patients were unresponsive to radioiodine and could be classified RAI‐refractory DTC according to the 2015 ATA guidelines.^21^ For large bone metastasis at certain sites in confined spaces such as cranium or spine, RAI treatments should be administrated cautiously, it may lead to compressive symptoms due to the possible enlargement of the tumor lesions induced by TSH elevation after hormone withdrawal or administration of rhTSH before I‐131 therapy.^22^ According to current treatment guidelines, when a patient with DTC is classified as refractory to RAI, further RAI treatment should be avoided.^21^

## 5 CONCLUSION

In summary, small proportion of follicular thyroid carcinoma manifests primarily as bone metastasis, its main symptom is localized pain, clinician should be cautious in eliciting the clinical history and these symptoms in middle age group, further imaging examination need to be carried out. We believe ultrasound‐guided core needle biopsy combined with IHC and molecular testing could determine the source and nature of the tumor, improve the diagnostic accuracy, help to predict distant metastasis and prognosis. Some bone metastases unresponsive to radioiodine classified RAI‐refractory after I‐131 therapy, according to current treatment guidelines, further RAI treatment should be avoided.

## AUTHOR CONTRIBUTIONS

**Zhiyuan Li:** Conceptualization; investigation; visualization; writing – original draft; writing – review and editing. **Jianbin Su:** Resources; supervision. **Jinjing Wang:** Investigation; resources. **Li Yan:** Project administration. **Huiqiang Zhang:** Visualization. **Xinyu Li:** Writing – original draft. **Yanhong Tai:** Resources; visualization. **Yi Fang:** Resources; supervision. **Tao Yan:** Supervision; writing – review and editing.

## FUNDING INFORMATION

No financial support was received for this study.

## CONFLICT OF INTEREST STATEMENT

The authors declare that they have no competing interests.

## ETHICS STATEMENT

This research was approved by The Medical Ethic Committee, the Chinese PLA General Hospital, Number: KY‐2024‐1‐1‐1.

## CONSENT

Written informed consent was obtained from the patient to publish this report in accordance with the journal's patient consent policy.

## Data Availability

The data that support the findings of this study are available on request from the corresponding author. The data are not publicly available due to privacy or ethical restrictions.
